# A Novel Modulator of the Renin–Angiotensin System, Benzoylaconitine, Attenuates Hypertension by Targeting ACE/ACE2 in Enhancing Vasodilation and Alleviating Vascular Inflammation

**DOI:** 10.3389/fphar.2022.841435

**Published:** 2022-03-11

**Authors:** Qi-Qiang Zhang, Feng-Hua Chen, Fei Wang, Xue-Mei Di, Wei Li, Hai Zhang

**Affiliations:** ^1^ Shanghai First Maternity and Infant Hospital, School of Medicine, Tongji University, Shanghai, China; ^2^ School of Pharmacy, Shandong University of Traditional Chinese Medicine, Jinan, China

**Keywords:** benzoylaconitine, antihypertensive effect, ACE, ACE2, renin–angiotensin system

## Abstract

The monoester alkaloids in *Aconitum carmichaelii*, including benzoylaconitine (BAC), benzoylmesaconine, and benzoylhypaconitine, were found to have anti-hypertensive effects in spontaneously hypertension rats (SHRs), of which BAC is the strongest. However, its antihypertensive target and underlying molecular mechanisms remain unclear. In this study, first, we screened the antihypertensive targets of BAC by using the CVDPlatform (www.cbligand.org/CVD) and found that ACE/ACE2 are the most possible targets. Then, we verified the effect of BAC on ACE/ACE2 by virtual docking, SPR, enzyme activity assay, and HUVECs cell experiment. We found that BAC could bind with ACE/ACE2, inhibit ACE activity and protein expression, and activate ACE2 enzyme activity. Using vascular function test *in vitro,* we found that BAC could target ACE/ACE2 to enhance endothelium-dependent vasorelaxation. In BAC-treated SHRs, the levels of ACE and AngII in serum were reduced while Ang (1–7) was increased significantly, and the expression of ACE was reduced, which suggested that BAC can inhibit ACE and activate ACE2 to inhibit AngI to AngII and promote AngII to Ang (1–7) to inhibit vasoconstriction and finally attenuate hypertension. Furthermore, the signaling pathways with regard to vasorelaxation and vascular inflammation were investigated. The results showed that BAC could significantly activate Akt/eNOS, increase NO production, and promote endothelial-related vasodilation; BAC could also reduce inflammatory factors TNF-α and IL6, inhibition of COX-2 expression, and IKB-α phosphorylation to reduce vascular inflammation in SHRs. In brief, BAC targets ACE/ACE2 to enhance endothelium-dependent vasorelaxation and reduce vascular inflammation to attenuate hypertension as a potential modulator of the renin–angiotensin system.

## Introduction

Hypertension is a high-risk chronic disease and is the leading cause of death globally, accounting for 10.4 million deaths per year worldwide (GBD 2017 Risk Factor Collaborators, 2018). It manifests primarily as high blood pressure and other cardiovascular complications, such as cardiovascular, neural, and renal diseases (Chockalingam, 2008). Drug therapy is the main treatment for hypertension. Besides regular medicines, angiotensin-converting enzyme inhibitors (ACEI), Ang II receptor blockers (ARB), calcium blockers, diuretics, and so on (Chockalingam, 2008), many traditional Chinese herbs have been used to clinically treat hypertension, especially *Aconitum carmichaelii*, which is officially recorded in Chinese pharmacopoeia and has been used to treat cardiovascular disease for many years.

*A. carmichaelii* is also known as “Fu Zi” sourced from the lateral root of *A. carmichaelii Debx*. It is beneficial for combating cardiovascular diseases, such as hypertension and heart failure (Xu et al., 2021). Alkaloids are considered the most predominant active ingredients in *A.* carmichaelii, which can be divided into three groups, including diester-diterpenoid alkaloids, monoester-diterpenoid alkaloids (MDAs), and alcohol-amine alkaloids. Some earlier studies demonstrated that diester-diterpenoid alkaloids, especially aconitine, are powerful agents for treating hypertension and contribute to their strong cardiovascular effects (Wang et al., 2020b). Unfortunately, they are not considered for clinical use owing to their toxic effects (Lin et al., 2004). In our previous study, systematic analysis of alkaloids using ultra-high-performance liquid chromatography-tandem mass spectrometry (UHPLC-MS/MS) revealed that diester-diterpenoid alkaloids could be transformed to MDAs by boiling or metabolism *in vivo*, and MDAs mainly contain benzoylaconitine (BAC), benzoylmesaconine (BMC), and benzoylhypaconitine (BHC) (Zhang et al., 2014; Zhang H. et al., 2015). Pharmacokinetic analysis in our previous study indicated that these MDAs had a low *T*_max_ value and high *t*_1/2_ (Zhang W. et al., 2015; Zhang et al., 2016b), which indicated that they can be absorbed rapidly and metabolized or excreted slowly. In addition, BAC was also found to be safer as its oral median lethal dose (*LD50*) is 1,500 mg kg^−1^ in mice (Wada et al., 2005). According to this evidence, we conferred that MDAs are potential therapeutic drugs for anti-hypertension.

However, the anti-hypertensive effect and targets of three MDAs are still unclear. In our previous study, we constructed the CVD intelligent analysis platform (www.cbligand.org/CVD) for the target screening and verification of anti-hypertensive compounds of traditional Chinese medicinal herbs (Zhang et al., 2016a). This platform involved main targets of the coagulation system, RAS system, adrenaline system, HMGCoA system, etc. We have successfully used the CVDPlatform to screen the active components and possible targets of a traditional Chinese herbal formula (Wang et al., 2020a).

Based on our preliminary study on the antihypertensive effect of three MDAs, we have carried out our research from the following aspects in this study: (1) evaluate the anti-hypertensive action of MDAs in spontaneously hypertension rats (SHRs) by i.v. injection and oral administration, and verify that BAC is the best one; (2) screen and identify the targets of BAC for anti-hypertension in the CVDPlatform; (3) verify the binding and effect between the BAC and its targets *via* virtual dock, SPR assay, and enzyme activity test; (4) verify the role of BAC on its targets in HUVECs; (5) verify the effect of BAC for anti-hypertension in SHRs; and (6) clarify the molecular mechanism by investigating the signaling pathways with regard to vasorelaxation and vascular inflammation. We hope that this study could provide support for drug discovery and clinical therapy of BAC for anti-hypertension.

## Materials and Methods

### Animal Model and Ethics Statement

This study was performed in accordance with the Chinese legislation and regulations of Laboratory Animals of the Chinese Animal Welfare Committee. The protocols for this study were approved by the ethics committee of the Tongji University School of Medicine (Shanghai, China, No. TJBG00121313) and authorized by the Shanghai First Maternity and Infant Hospital, Tongji University School of Medicine (Shanghai, 201204, China).

Fifteen-week-old male SHRs (280.0 ± 10 g) and 10-week-old male Sprague–Dawley (SD) rats (310.0 ± 10 g) were obtained from Charles River Co., Ltd. (Beijing, China). The animals were housed at the Tongji University Animal Center for a week for acclimatization, where they were exposed to a 12-h light/12-h dark cycle under conditions of controlled temperature of 25 ± 2°C and relative humidity of 45% ± 5%. All rats were provided access to standard rat chow and water *ad libitum*. This protocol was approved by the University of Tongji Animal Welfare Committee in accordance with the guidelines issued by the China Council on Animal Care and adhered to the *Guide for the Care and Use of Laboratory Animals* published by the United States National Institutes of Health.

### Surgical Procedure

The SHRs were surgically implanted with a catheter *via* the left femoral artery, vein, and stomach after anesthetizing with 2% pentobarbital sodium (40 mg kg^−1^ i.p.,). A pressure sensor was inserted into the terminal of the arterial catheter and connected to a PowerLab system (ADInstruments, New South Wales, Australia) for real-time blood pressure monitoring.

### Dosage Information

All three MDAs (BAC, BMC, and BHC) were purchased from Nature-Standard Co., Ltd. (Shanghai, China) and their purities were all over 98%. Information on the three MDAs is shown in Table 1. The anti-hypertensive effects of MDAs were studied in both acute and chronic experiments. The 16-week-old SHRs were used in animal experiments, and each group had six rats. In the acute experiment, drug administration was *via* i.v. injection. For i.v. injection, the animals were randomly assigned into four groups: vehicle (0.1% DMSO–0.9% salt solution, *n* = 6), low-dose group (0.6 mg kg^−1^ body weight, *n* = 6), medium-dose group (2 mg kg^−1^ body weight, *n* = 6), and high-dose group (6 mg kg^−1^ body weight, *n* = 6). The drug was administered using a venous catheter. In chronic experiments, the animals were randomly assigned into five groups: vehicle (5% Tween 80–0.9% salt solution, *n* = 6), captopril group (5 mg kg^−1^ body weight, *n* = 6), low-dose group (3 mg kg^−1^ body weight, *n* = 6), medium-dose group (10 mg kg^−1^ body weight, *n* = 6), and high-dose group (30 mg kg^−1^ body weight, *n* = 6). Oral administration with drug or 5% Tween 80–0.9% salt solution was carried out once a day, and continued for 14 days. The drugs were dissolved in 0.1% DMSO–0.9% salt solution for intravenous injection and in 5% Tween 80–0.9% salt solution for oral administration. Blood pressure was recorded in real time.

**TABLE 1:** Information on BAC, BHC, and BMC.

Full name	Abbreviation	Formula	MW (g/mol)
Benzoylaconitine	BAC	C_32_H_45_NO_10_	604
Benzoylmesaconine	BMC	C_31_H_43_NO_10_	590
Benzoylhypaconitine	BHC	C_31_H_43_NO_9_	574

### Blood Pressure Recording

Real-time blood pressure level was monitored after drug treatment in the acute and chronic experiments. All animals were surgically implanted with a catheter in the left femoral artery. After connection to the monitor, all animals were placed at room temperature of 25°C for 0.5–1 h and the average blood pressure value was calculated based on a real-time blood pressure curve (10 s of every 30 s).

### Target Screening, Virtual Docking, and Surface Plasmon Resonance Assay

To screen the potential targets of the MDAs in hypertension, a CVDPlatform (www.cbligand.org/CVD) was used as previously reported (Zhang et al., 2016a). The results of database screening were verified by virtual docking with Discovery Studio 3.5 (BIOVIA, San Diego, CA, United States) and then validated in the SPR assay. In the SPR assay, a solution of protein standards was prepared, and the concentration of recombinant human ACE (rhACE) protein and rhACE2 (Proteintech, Rosemont, IL, United States) solution was set to 500 μg ml^−1^. After combining with the chips, a series of concentrations of compounds, which were double-diluted at each step with 5% DMSO–1.05 × phosphate buffered saline (PBS) solution, were tested for their binding affinity to the proteins. Finally, the level of binding between the proteins and each molecule was determined based on the *K*_d_ value.

### Enzyme Kinetics Study

ACE and ACE2 activities were measured using the ACE activity assay kit and SensoLyte390 ACE2 activity assay kit (Anaspec, Fremont, CA, United States), respectively, according to the manufacturer’s instructions with some modifications. Briefly, rhACE solution dissolved in deionized water was used for analysis. ACE activity was measured in a reaction system with 50 μl of the sample and 50 μl of the ACE substrate solution with or without BAC (10^–4^ to 10^–2^ μM). ACE2 activity was assayed in the same manner as ACE activity. All assays were performed every 10 s for 30 min at 37°C using a Varioskan LUX fluorescence microplate reader (Thermo Fisher Scientific, Waltham, MA, United States). The autofluorescence value in each assay was subtracted from the measured values to generate the final results. The relative fluorescence unit of each sample was normalized to the corresponding total protein concentration, which was measured using a Pierce BCA protein assay kit (Thermo Fisher Scientific).

### Cell Culture and Treatment

Human umbilical vein endothelial cells (HUVECs) were isolated from umbilical cords as previously described (Jaffe et al., 1973). They were purchased from ATCC (cat# CRL-1444, Manassas, VA, United States) and cultured in endothelial cell medium (ECM, ScienCell, Carlsbad, CA, United States) supplemented with 5% FBS, 1% endothelial cell growth supplement, and 1% antibiotics (Sigma, St. Louis, MO, United States). Cells harvested between the third and tenth passages were used in the experiments. Cells were cultured until they were ∼80% confluent. After pre-starving in a quiescing ECM supplemented with 1% FBS and 1% antibiotics for 24 h, the cells were treated using different concentrations of BAC (Control, DMSO, 25, 50, and 100 μM, dissolved in 0.1% DMSO–DMEM) with or without MG132, an inhibitor of proteasome, at 20 nM or 40 nM for 24 h. Then, cell samples were collected for Western blot analysis or immunofluorescence staining.

### Drug Cytotoxicity

The potential of cells to maintain or recover viability after treatment with each agent was determined. Cell viability can be distinguished from the all-or-nothing states of life and death using a quantifiable index between 0 and 1 (or 0 and 100%). Cell viability was measured by a Cell Counting Kit-8 (CCK-8) Assay Kit (EnoGene, Nanjing, China). The cells (1 × 10^4^) were seeded in a 96-well plate. After reaching 90% density and starving for 24 h, the cells were treated with different concentrations of BAC (0, 12.5, 25, 50, 100, 200, and 400 μM) or with 0.1% DMSO for 48 h. CCK-8 reagent (10 μl) was added to fresh medium (100 μl) in each well. After 1 h of incubation, the absorbance was measured at 450 nm using a microplate reader (Thermo Fisher Scientific). The measurement of cell viability at each concentration was repeated three times.

### Immunofluorescence Staining

After treatment with different concentrations of BAC (Control, DMSO, 25, 50, and 100 μM, dissolved in 0.1% DMSO–DMEM) for 24 h, the cells were washed with PBS three times, fixed in 4% paraformaldehyde for 20 min, permeabilized with 0.3% Triton X-100 for 20 min, and then blocked with 5% BSA for 30 min at room temperature of 25°C. Next, the cells were treated with primary antibodies against ACE (1:100, ABclonal) and ACE2 (1:100, ABclonal) at 4°C overnight, followed by treatment with fluorescein isothiocyanate-labeled secondary antibody (1:400, Beyotime, Wuhan, China) for 1 h at room temperature (25°C). 4′,6-Diamidino-2-phenylindole dihydrochloride (DAPI, Beyotime) was used for nuclear staining. The samples were analyzed using a confocal laser scanning microscope (Zeiss LSM 510 META, Oberkochen, Germany).

### Histological and Immunofluorescence Staining Analysis

After euthanizing the rats with sodium pentobarbital (100 mg kg^−1^, i.p.) administration in combination with isoflurane inhalation, the aortas were harvested directly according to a previously described method. The aortas were fixed in 4% paraformaldehyde for 24 h and embedded in paraffin. The cross-sections (5 μm) were stained with hematoxylin and eosin or subjected to immunostaining. Immunofluorescence staining for ACE and ACE2 was performed according to the manufacturer’s instructions (Servicebio, Wuhan, China).

### Western Blot Analysis

The cell samples and tissue sample were collected and placed in ice rapidly. All samples were washed with PBS and lysed in a buffer containing protease inhibitors (Roche, Basel, Switzerland). Total proteins were extracted after incubation in lysis buffer for 15 min on ice. The supernatants were collected after centrifugation (12,000 × *g*, 4°C, 10 min). Mitochondrial proteins were isolated using a mitochondrial isolation kit (YEASEN, Shanghai, China). Protein concentration in cell lysates was measured using a Bio-Rad DC Protein Assay Kit (Pierce, Rockford, United States). The proteins (20 μg) were separated by electrophoresis at 100 V for 80 min in a 10% polyacrylamide gel and electrotransferred (Bio-Rad) to polyvinylidene fluoride membranes at 110 V for 70 min. To prevent non-specific binding, the membranes were blocked by treating with 5% milk for 60 min at 20°C. The proteins were treated with primary antibodies against ACE (1:1,000; cat#A2805, ABclonal, Shanghai, China), ACE2 (1:1,000; cat#A4612, ABclonal), eNOS (1:1,000; cat#A20985, ABclonal), p-eNOS^Ser1177^ (1:1,000; cat#AP0515, ABclonal), COX-2 (1:1,000; cat#A3560, ABclonal), IKB-α (1:1,000; cat#A11397, ABclonal), p-IKB-α (1:1,000; cat#AP0707, ABclonal), and GAPDH (1:10,000; cat#AC027, ABclonal) at 4°C overnight, and were then treated with HRP-secondary antibodies (1:15,000; ABclonal) at 25°C for 1 h. After washing three times with TBS-Tween (for 5 min), the membrane was observed.

### Plasma Biomarker Analysis

Blood samples were collected from the left femoral artery of rats at the end point, followed by centrifugation at 1,000 × *g* for 15 min at 4°C. Only serum was collected and stored at −80°C until analysis. The concentrations of circulating ACE, ACE2, Ang I, Ang II, Ang (1–7), TNF-α, and IL-6 in serum were measured using the ELISA kits [ACE, cat#xy-R2401c, X-Y Biotechnology, Shanghai, China; ACE2, cat#xy-ACE2-Ra, X-Y Biotechnology; Ang I, cat#xy-E12541, X-Y Biotechnology; Ang II, cat#xy-R1430c, X-Y Biotechnology; Ang (1–7), cat#xy-Ang1-7-Ra, X-Y Biotechnology; TNF-α, cat#GM1149, Servicebio, Shanghai, China; IL-6, cat#GM1154, Servicebio] according to the manufacturer’s instructions. The absorbance was measured using a microplate reader (Thermo Fisher Scientific) at 450 nm and calculated according to the standard curve. Each experimental group had three duplicate wells, and the experiment was repeated three times.

### Vascular Function

Endothelium-dependent vasorelaxation using aorta was evaluated as previously described (Liao et al., 2019). Briefly, after euthanizing the SD rat with sodium pentobarbital (100 mg kg^−1^, i.p.) administration in combination with isoflurane inhalation, thoracic aorta (diameter: 150–250 μm, length: 2 mm) was mounted on two tungsten wires and attached to a tension sensor system (ADInstruments). After balancing for 2 h in Krebs’ solution at 37°C, arteries were exposed to 10 µM of phenylephrine (PE, Sigma-Aldrich, St. Louis, MO, United States) twice, followed by a single dose of acetylcholine (Ach, 3 μm, Sigma-Aldrich, St. Louis, MO, United States) to evaluate the integrity of the vessel. Afterward, the vessel was pre-constricted to maximum using AngI (Sigma-Aldrich) at 10^–2^ μM and then treated with a series concentration of BAC or A779 (Sigma-Aldrich) or captopril at 10^–6^ to 10^–1^ μM. Lastly, a cumulative concentration response curve to BAC with A779 (at 10^–2^ μM) was performed to assess the endothelium-dependent vasorelaxation.

### Measurement of Total NO

Total NO levels in serum obtained from SHRs treated with vehicle, BAC, or captopril for 14 days were quantified by using total nitric oxide assay kit (Beyotime, Shanghai, China) as described previously. The absorbance was measured using a microplate reader (Thermo Fisher Scientific) at 540 nm and calculated according to the standard curve. Each experimental group had three duplicate wells, and the experiment was repeated three times.

### Data and Statistical Analysis

Data are expressed as mean ± SEM. Differences between two groups were compared using a two-tailed Student’s *t*-test. Differences among multiple groups were analyzed using two-way ANOVA followed by Bonferroni’s post-hoc test. Differences with *p* < 0.05 were considered statistically significant.

## Results

### MDAs Attenuated High Arterial Pressure in SHRs

**FIGURE 1:**
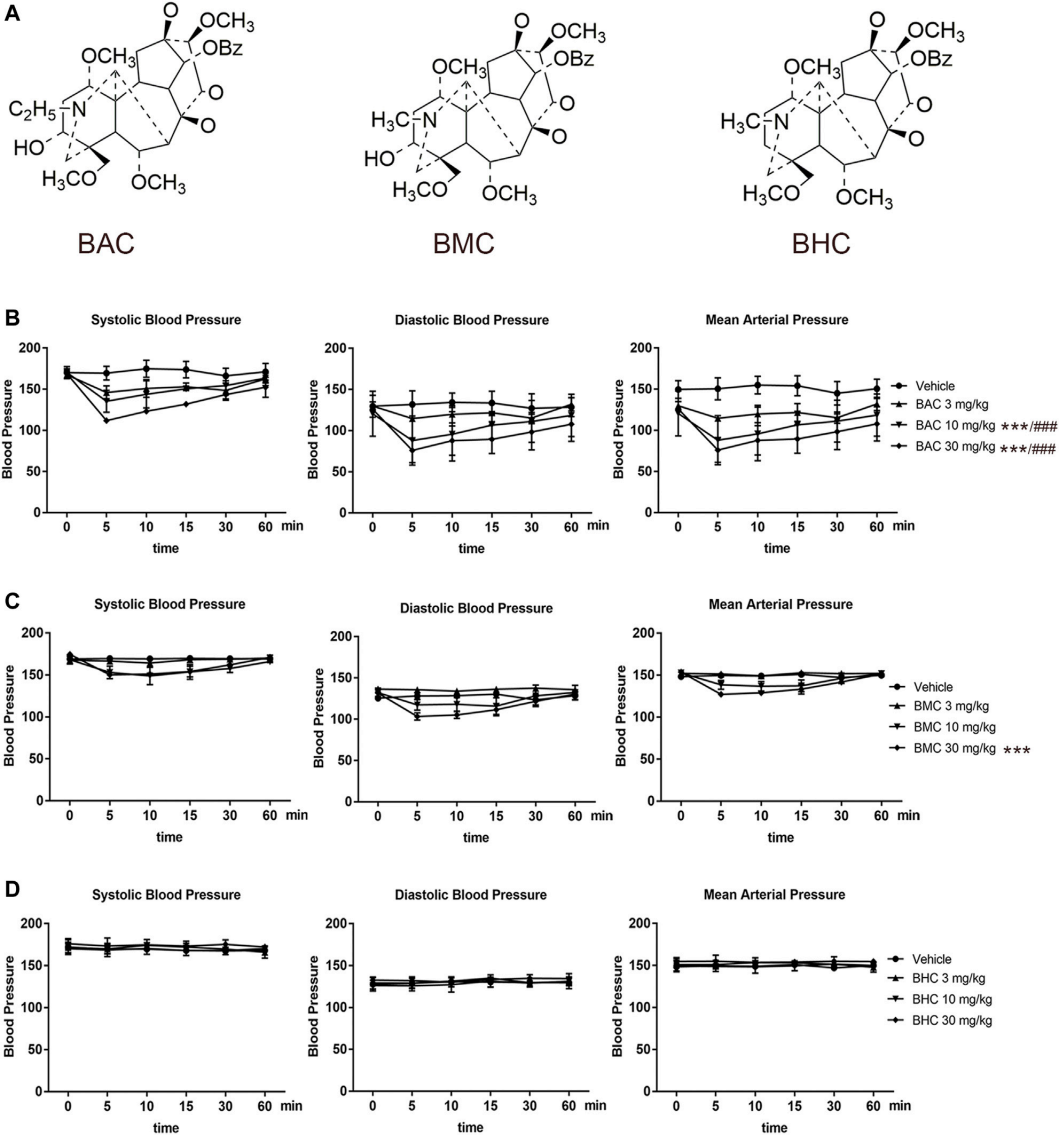
Anti-hypertensive effect of three monoester alkaloids (MDAs). **(A)** Structure of MDAs. Left to right were benzoylaconitine (BAC), benzoylmesaconine (BMC), and benzoylhypaconitine (BHC). **(B–D)** Blood pressure measurement and heart rate recording after intravenous administration of drugs in spontaneous hypertension rats (SHRs). SHRs were divided into four groups randomly. The experimental groups were the vehicle group (*n* = 6), the low-dose group (0.6 mg kg^−1^, *n* = 6), the medium-dose group (2 mg kg^−1^, *n* = 6), and the high-dose group (6 mg kg^−1^, *n* = 6). **(B)** To BAC, **(C)** to BMC, and **(D)** to BHC. The experimental results were expressed by mean ± SEM, *p < 0.005, as compared with the vehicle control, ###*p* < 0.005, as compared with the BMC or BHC in the same dosage.

The structures of the three MDAs are shown in Figure 1A. We determined whether they elicited anti-hypertensive effects *via* experiments on rats with acute and chronic hypertension by assessing systolic blood pressure (SBP), diastolic blood pressure (DBP), mean arterial pressure (MAP), and heart rate (HR). In the acute experiment, we observed that BAC and BMC exerted observable anti-hypertensive effects but BHC did not. Even at the lowest dose (0.6 mg kg^−1^), BAC administered *via* i.v. injection for 1–5 min could lower SBP, DBP, and MAP by more than 20 mmHg, with the anti-hypertensive effect lasting for at least 30 min. At a medium dose (2 mg kg^−1^) and high dose (6 mg kg^−1^), BAC had a better effect (Figure 1B). The effect of BMC was weaker than that of BAC as blood pressure was reduced by approximately 20 mmHg only at a dosage of 6 mg kg^−1^ (Figure 1C). BHC at a dosage lower than 6 mg kg^−1^ exerted no anti-hypertensive effects (Figure 1D). Heart rate had no significant change after drug administration. Thus, BAC is the best candidate drug for lowering blood pressure among these MDAs. The results showed that at medium and high dosage, BAC and BMC exerted anti-hypertensive effects in a dose-dependent manner.

### MDAs Bound With ACE and ACE2 in Virtual Docking and SPR Assay

**FIGURE 2:**
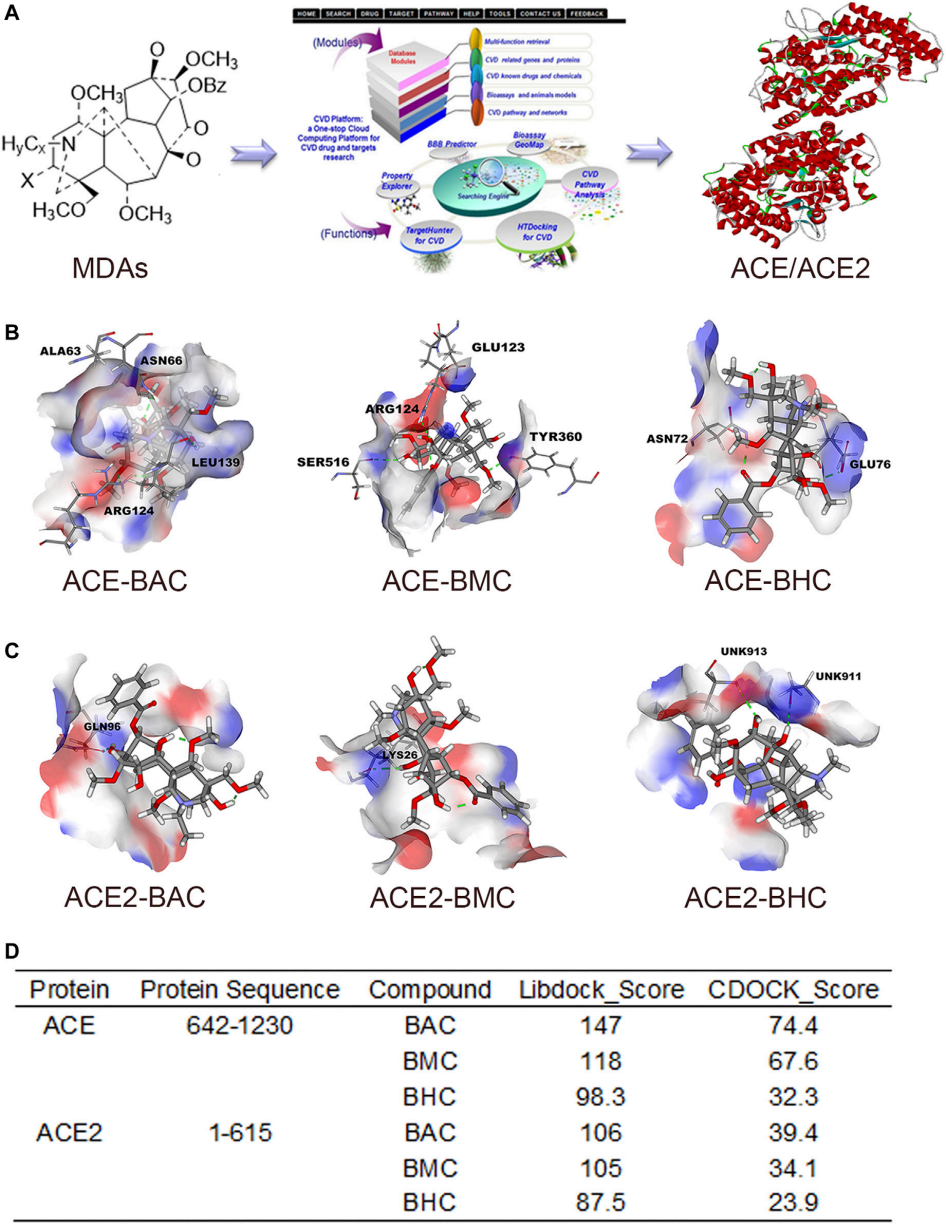
MDAs bound with ACE and ACE2 in Virtual Docking. Target screening and virtual docking of MDAs. **(A)** Drug molecular potential target screening based on the CVD-Platform online analysis platform. **(B,C)** Molecular docking of MDAs at ACE and ACE2 active sites. Drug molecules interact with amino acid residues of ACE and ACE2 mainly through hydrogen bonds. Blue: donor; Red: acceptor. **(D)** Docking results of MDAs with ACE and ACE2. The docking modes including Libdock (high throughput) and CDOCK (precision docking).

After screening in the CVDPlatform, ACE and ACE2 were found to be potential targets of the three MDAs (Figure 2A). To verify the binding relationship of MDAs with ACE and ACE2, virtual docking (a method in virtuality) was used. In virtual docking, the Libdock results of BAC, BMC, and BHC were 147, 118, and 98.3 with ACE and 106, 105, and 87.5 with ACE2. The CDOCK results of BAC, BMC, and BHC were 74.4, 67.6, and 32.3 with ACE and 39.4, 34.1, and 23.9 with ACE2, respectively. Comparing the dock score of each molecule and protein, BAC and BMC were found to have a better binding relationship with ACE owing to hydrogen bonding (Figure 2B), whereas their binding relationship with ACE2 was ambiguous (Figures 2C,D). BHC appeared to have the weakest binding relationship among these compounds. In summary, among these three MDAs, BAC showed the best binding with both ACE and ACE2, followed by BMC and BHC.

### MDAs Bind With ACE/ACE2 and Affected Their Activity Under Cell-Free Conditions *In Vitro*

**FIGURE 3:**
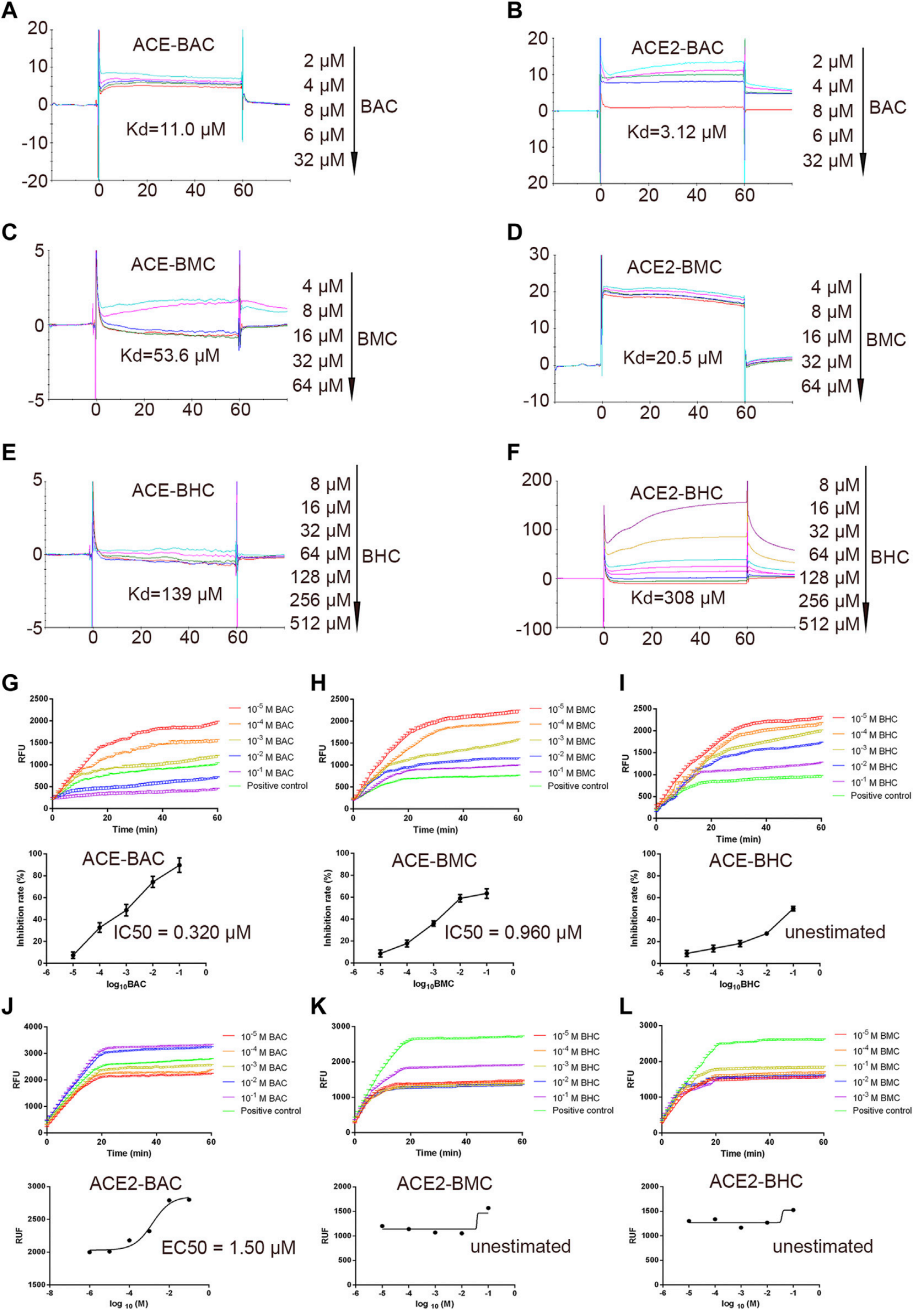
*In vitro* binding and bioactivity verification of MDAs with proteins ACE and ACE2 under cell-free conditions. **(A–F)** Binding verification between protein and molecules were measured by SPR. A series of concentration step of drug diluted with 5% DMSO–1.05*PBS was reacted with the protein in chip, the electrical signal value was recorded, and the dissociation constant *K*_d_ value was determined. **(A)** ACE with BAC, **(B)** ACE with BMC, **(C)** ACE with BHC, **(D)** ACE2 with BAC, **(E)** ACE2 with BMC, and **(F)** ACE2 with BHC. **(G–L)** Bioactivity verification of MDAs by enzyme kinetics experiment. Dose-dependent hrACE2 activity in the presence of MDAs. Then, MDAs of series of concentrations were added into the system respectively. Results were analyzed by GraphPad Prism 7.0 software. Each group was repeated three times. **(A)** ACE and BAC, **(B)** ACE and BMC, **(C)** ACE and BHC, **(D)** ACE2 and BAC, **(E)** ACE2 and BMC, and **(F)** ACE2 and BHC. The value was expressed by mean ± SEM.

The SPR assay was used to identify the binding between the drugs and proteins (Nguyen et al., 2015). The results showed that MDAs interacted with the rhACE protein with a *K*_d_ of 11.0, 53.6, and 139 µM (Figures 3A,C,E). They interacted with the rhACE2 protein with a *K*_d_ of 3.12, 20.5, and 308 µM (Figures 3B,D,F). These are consistent with the results of virtual docking. In order to further verify the effect of MDAs on the biological activities of ACE and ACE2 proteins, enzyme kinetics experiments were carried out. According to the experimental results, we observed that BAC and BMC clearly inhibited ACE activity, and their IC_50_ values were 0.320 and 0.960 μM, respectively (Figures 3G,H). However, BHC had little effect on ACE activity, and its IC_50_ value could not be determined (Figure 3I). Moreover, MDAs were found to have an excitatory effect on ACE2 activity. Among them, BAC had the most significant effect, and its EC_50_ value was 1.50 μM (Figure 3J), while BMC and BHC played weaker pharmacodynamics effects, and their EC_50_ values could not be determined (Figures 3K–L-L). Taken together, the results showed that BAC had the strongest effect on ACE and ACE2 under cell-free conditions.

### Benzoylaconitine Inhibited the Protein Expression of ACE Directly in HUVECs

**FIGURE 4:**
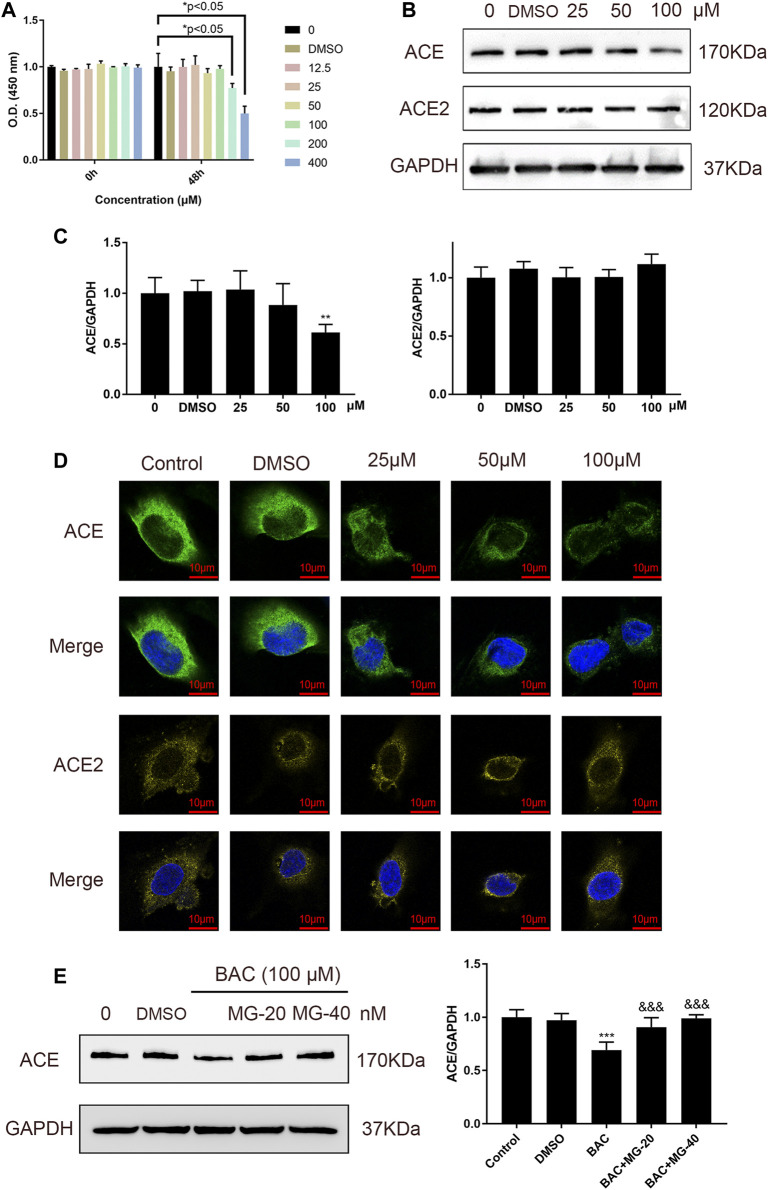
BAC inhibited ACE directly in HUVECs. **(A)** Starved HUVECs were preconditioned with a series of BAC (0, 12.5, 25, 50, 100, 200, and 400 μM) or with 0.1% DMSO for 48 h and then measured by CCK-8 assay (*n* = 3) at 450 nm. *p < 0.05 compared with 0 group. **(B,C)** After being starved, HUVECs were treated with serial concentrations of BAC (0, DMSO, 25, 50, and 100 μM) for 24 h to extract protein, and the protein quantity of ACE and ACE2 was detected by Western blot. The gray value of protein was determined by ImageJ. **(D)** Representative image of fluorescence immunoassay for protein expression and fluorescence localization of ACE and ACE2. Starved HUVECs were treated with serial concentrations of BAC (0, DMSO, 25, 50, and 100 μM) for 24 h and measured by fluorescence immunoassay. **(E)** After being starved, HUVECs were treated with BAC (100 μM) and with or without MG132 at 20 nM or 40 nM for 24 h. The protein quantity of ACE was detected by Western blot. The gray value of protein was determined by ImageJ. The value was expressed by mean ± SEM. **p < 0.05, ***p < 0.05, as compared with the 0 group.

Binding of BAC with ACE prompted us to examine whether BAC affects the expression of ACE and ACE2 in HUVECs, which are model cells for studying hypertension. Cell viability experiment was used to evaluate the toxicity of BAC on HUVECs, and the results showed that BAC at a dose lower than 100 μM had no significant effect (Figure 4A). The protein expression of ACE and ACE2 in RAS was measured by Western blotting and immunofluorescence staining, and we found that BAC can inhibit the expression of ACE, but does not affect the protein expression of ACE2 (Figures 4B–D). Adding proteasome inhibitor, MG132, we further found that BAC could promote ACE protein degradation to inhibit the expression level of ACE directly.

### Benzoylaconitine Attenuated Hypertension in SHRs

**FIGURE 5:**
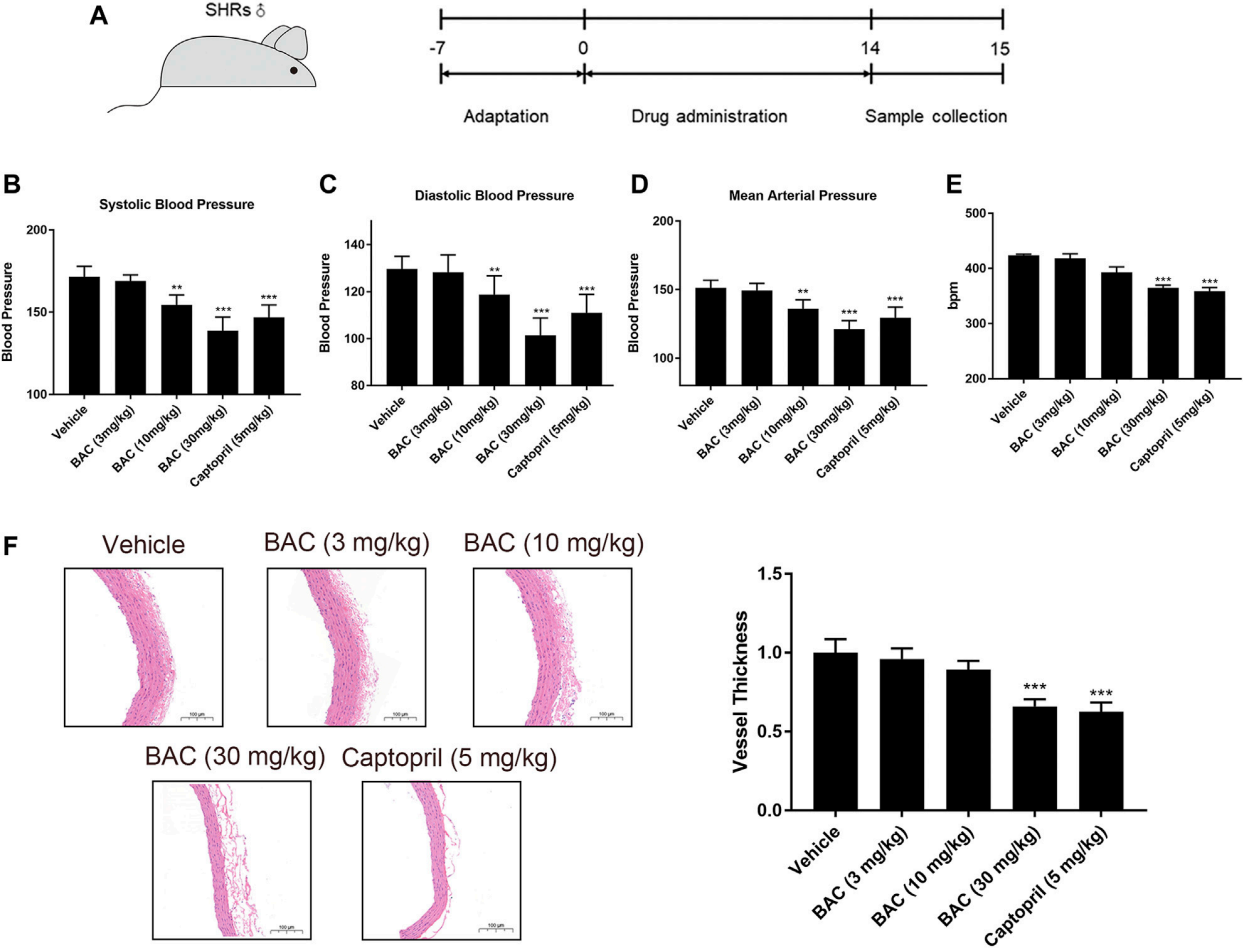
BAC attenuated hypertension in SHRs after a 14-day oral administration. The experimental groups were the vehicle group (*n* = 6), the low-dose group (3 mg kg^−1^, *n* = 6), the medium-dose group (10 mg kg^−1^, *n* = 6), the high-dose group (30 mg kg^−1^, *n* = 6), and the captopril group (5 mg kg^−1^, *n* = 6). All rats were given a 14-day oral administration. **(A)** The process of animal administration and sample collection in our research. **(B–D)** BAC reduced the blood pressure of SHRs measured after 14 days of oral administration. **(B)** Systolic blood pressure (SBP), **(C)** diastolic blood pressure (DBP), and **(D)** mean arterial pressure (MAP). **(E)** Heart rate recording. **(F)** Representative hematoxylin and eosin stain image of SHRs with or without BAC oral administration. Data represented as mean ± SEM from six animals per treatment group. **p < 0.05, ***p < 0.005, as compared with the vehicle control.

In view of the above results, BAC showed the most significant anti-hypertensive effect. Thus, a long-term animal experiment was conducted using BAC. Captopril (5 mg kg^−1^) was used as a positive control for this experiment. The process of this experiment is shown in Figure 5A. The SBP, DBP, and MAP of SHRs were 172 ± 6 mmHg, 130 ± 5 mmHg, and 151 ± 5 mmHg, respectively. After 14 days of oral administration, the SBP, DBP, and MAP reduced in the medium-dose group (10 mg kg^−1^) and the high-dose group (30 mg kg^−1^). The SBP, DBP, and MAP of the medium-dose group were 154 ± 6 mmHg, 119 ± 8 mmHg, and 136 ± 7 mmHg, respectively, and those of the high-dose group were 139 ± 6 mmHg, 101 ± 7 mmHg, and 121 ± 6 mmHg, respectively. However, the blood pressure of the low-dose group did not significantly differ from the vehicle group (Figures 5B–D). Heart rate changed in the high-dose group and captopril group, and they had similar heart rate changes, which means that BAC could also reduce the heart rate after long-term drug treatment (Figure 5E). The vascular smooth muscle was thicker in the absence of BAC treatment. This suggests that BAC could reverse smooth muscle remodeling to play an anti-hypertensive effect (Figure 5F).

### Benzoylaconitine Attenuated Hypertension Through Regulating RAS in SHRs

**FIGURE 6:**
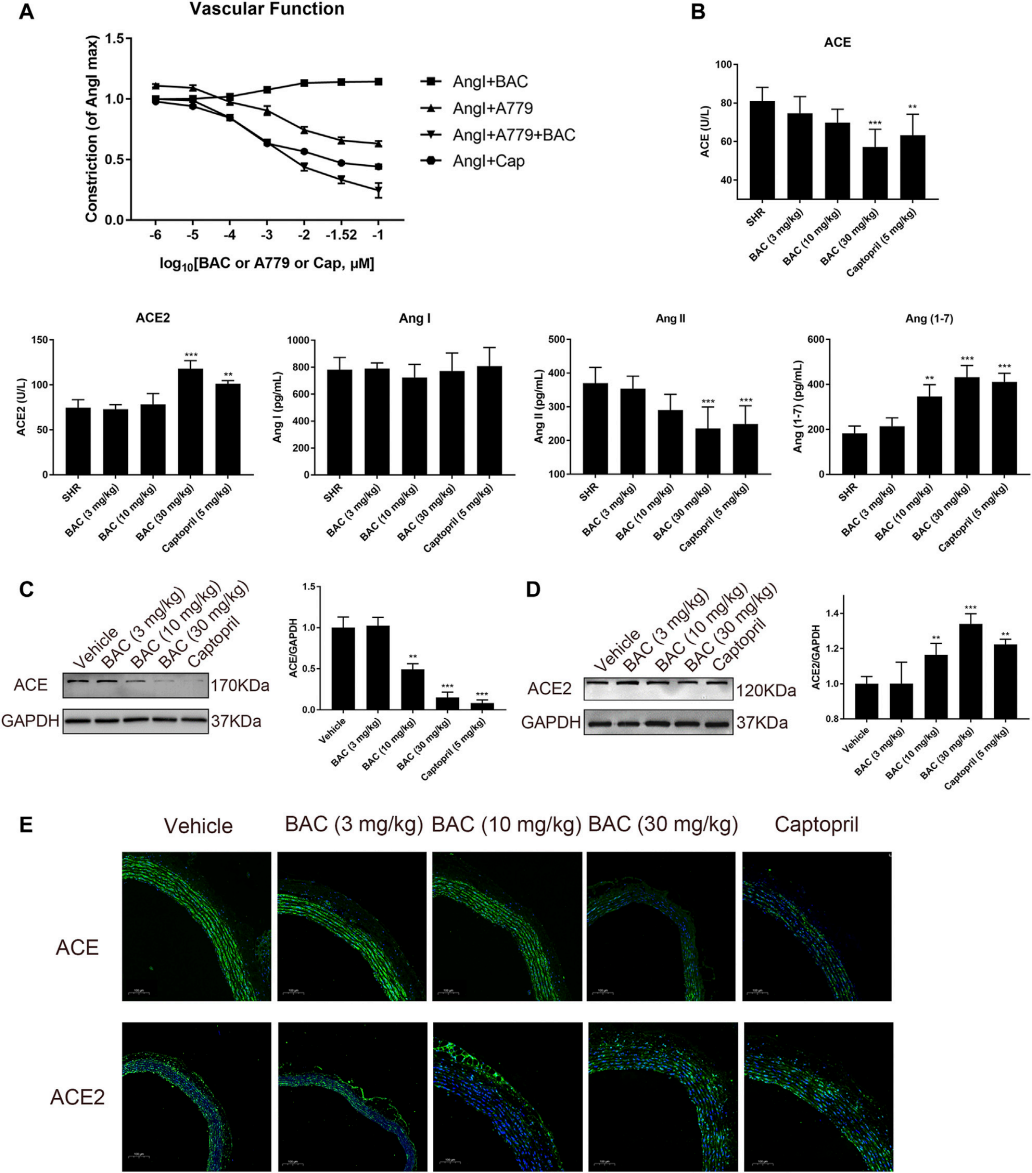
BAC attenuated vascular constriction and regulated the RAS in SHRs. **(A)** *Ex vivo* cumulative response curve was performed with isolated aorta. Tissue was isolated from SD rat and balanced for 2 h in Krebs’ solution at 37°C and then it was stimulated with Ang I at 10^–2^ μM and a series concentration of BAC or A779 or captopril at 10^–6^ to 10^–1^ μM. Abbreviations: Ang I + BAC: Ang I at 10^–2^ μM and a series concentration of BAC; Ang I + A779: Ang I at 10^–2^ μM and a series concentration of A779; Ang I + A779 + BAC: Ang I at 10^–2^ μM, A779 at 10^–1^ μM and a series concentration of BAC; Ang I + Cap: Ang I at 10^–2^ μM and a series concentration of captopril. **(B–E)** The experimental groups were the vehicle group (*n* = 6), the low-dose group (3 mg kg^−1^, *n* = 6), the medium-dose group (10 mg kg^−1^, *n* = 6), the high-dose group (30 mg kg^−1^, *n* = 6), and the captopril group (5 mg kg^−1^, *n* = 6). All rats were given a 14-day oral administration. **(B)** Plasma from the rats were collected at the end point to evaluate the levels of circulating, including ACE, ACE2, Ang I, Ang II, and Ang (1–7) concentration. **(C,D)** Total proteins were extracted from aorta of SHRs. Expressions of **(C)** ACE and **(D)** ACE2 were normalized to GAPDH. The gray value of protein blotting was measured by ImageJ. **(E)** Representative image of fluorescence immunoassay for protein expression and fluorescence localization of ACE and ACE2. The value was expressed by mean ± SEM. **p < 0.05, ***p < 0.05, as compared with the vehicle group.

Vascular function was performed in isolated vascular rings as in a previous study (Liao et al., 2019). The results demonstrated that BAC could attenuate Ang I-induced vascular constriction, and it could be abolished by Mas inhibitor, A779 (Figure 6A). The serological experiments related to the RAS were carried out in this study. The results showed that the levels of ACE and Ang II decreased, the levels of Ang (1–7) significantly increased after BAC treatment, but that of Ang I and ACE2 did not change in the blood of SHRs (Figure 6B). The effect of BAC on RAS protein expressions was also determined in SHRs after 14 days of BAC oral administration by Western blotting and tissue immunofluorescence. We found that BAC significantly downregulated ACE expression, while it had no effect on ACE2 expression in the aorta (Figures 6C–E). These results suggested that BAC could not only inhibit the activity and protein expression of ACE but also activate the ACE2 activity in a dose-dependent manner.

### Benzoylaconitine Activated Endothelium-dependent Vasorelaxation and Reduced Vascular Inflammation

**FIGURE 7:**
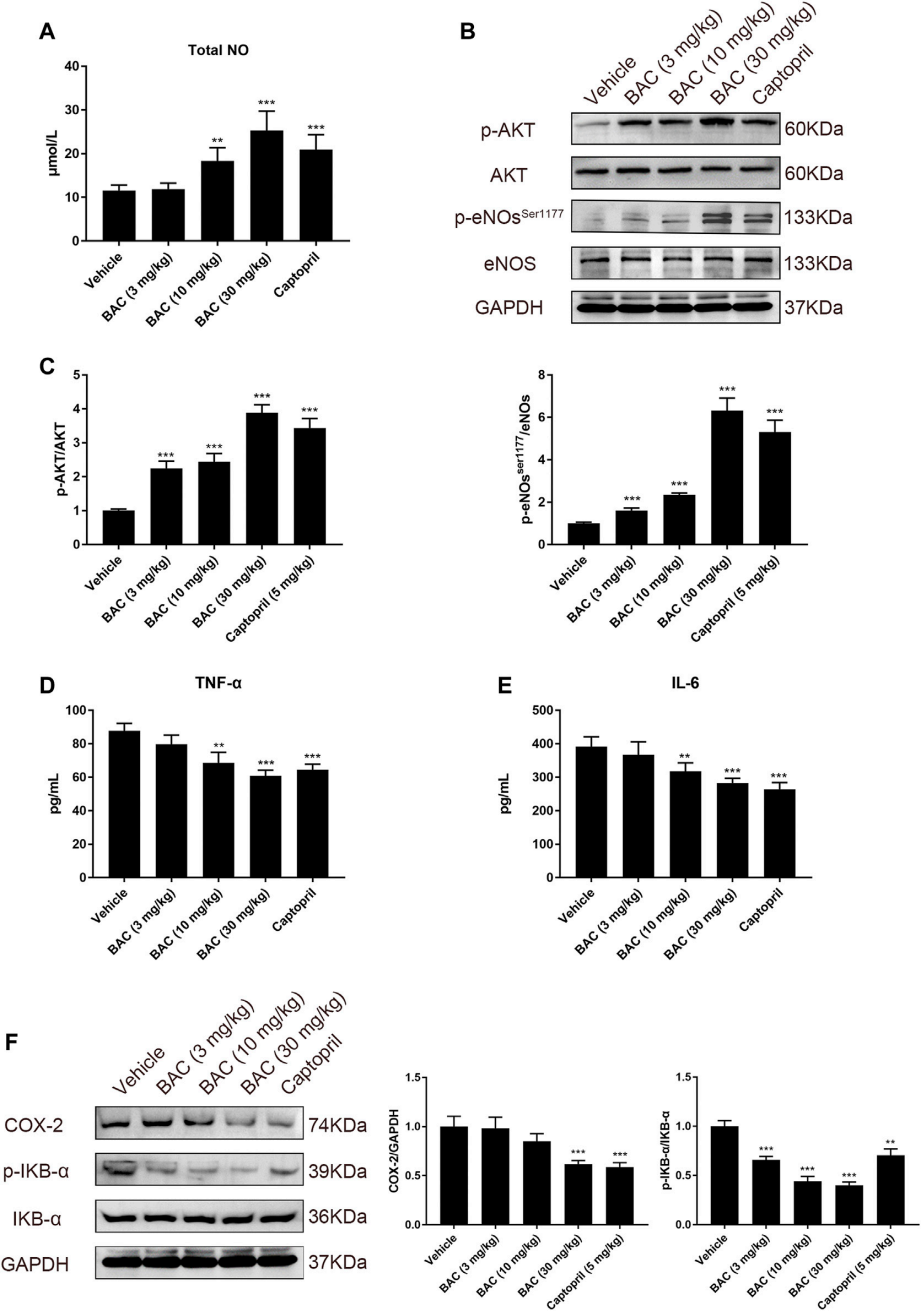
BAC activated the NO system and reduced vascular inflammation in SHRs. The experimental groups were the vehicle group (*n* = 6), the low-dose group (3 mg kg^−1^, *n* = 6), the medium-dose group (10 mg kg^−1^, *n* = 6), the high-dose group (30 mg kg^−1^, *n* = 6), and the captopril group (5 mg kg^−1^, *n* = 6). All rats were given a 14-day oral administration. Serum and tissues were collected at the end point. Total proteins were extracted from aorta of SHRs. **(A)** Total NO in serum was measured by total nitric oxide assay kit at 540 nm. **(B,C)** Protein quantities of p-Akt, Akt, eNOs, and p-NO^ser1177^ were detected by Western blot, and gray values were quantified by ImageJ. **(D,E)** The TNF-α and IL-6 in serum were measured by a microplate reader at 450 nm. **(F)** Protein quantities of COX-2, p-IKB-α, and IKB-α were detected by Western blot, and gray values were quantified by ImageJ. The value was expressed by mean ± SEM. ***p < 0.05, as compared with the vehicle group.

Concentration of total NO in serum was measured in SHRs, and the results suggested that BAC could increase NO level in a dose-dependent manner in SHRs (Figure 7A). In the vessel of SHRs, we assessed the protein expression and phosphorylation of eNOS in SHRs by Western blotting. As anticipated, the p-Akt and p-eNOS^Ser1177^ were activated, which indicated NO release in SHR tissue (Figures 7B,C). We further found that BAC reduced the level of TNF-α and IL-6 in serum and downregulated expression of COX-2 and phosphorylation of IKB-α (Figures 7D–F), which were markers of vascular inflammation. These results suggested that BAC targeted the ACE/ACE2 to modulate the vasorelaxation and vascular inflammation.

## Discussion

Natural products such as herbal medicine are abundant sources of compounds for drug discovery for anti-hypertension. The MDAs, a series of natural products derived from “Fu Zi”, have potential protective effects against hypertension (Zhang et al., 2016a; Zhang et al., 2016b). However, the systematic evaluation of the blood-pressure-lowering effects of MDAs, the study of their targets, and pharmacological mechanism are still incomplete.

In this study, we used a real-time blood pressure monitoring system in MDA-treated SHRs to evaluate their effect. We found that BAC is the most effective in lowering blood pressure among the MDAs. The CVDPlatform is a very effective intelligent target screening platform that we previously built (Zhang et al., 2016a). In this study, we screen the potential targets of BAC in the CVDPlatform and found that ACE/ACE2 are the potential targets for anti-hypertension. We further validated their binding and activities by using virtual dock, SPR, and enzyme activity assays under cell-free conditions and found that BAC could bind with ACE/ACE2, inhibit ACE activity, and activate ACE2 activity in a dose-dependent manner. In BAC-treated HUVECs, we investigated the protein expression of ACE/ACE2 and found the BAC directly inhibited the protein expression of ACE by promoting its degradation and had no effect on the protein expression of ACE2. In the BAC-treated isolated vessel, we found that BAC could inhibit Ang I-induced vasoconstriction and promote ACE2-related vasorelaxation. In long-term BAC-treated SHRs, BAC significantly lowered blood and heart rate, and reduced vessel thickness. Furthermore, BAC reduced the level of ACE and Ang II, increased the level of Ang (1–7), and had no effect on the level of ACE2 in blood circulation. In SHR vessel tissue, BAC inhibited the protein expression of ACE and had little effect on ACE2. ACE and ACE2 are the main regulatory enzymes in RAS and the key targets for anti-hypertension (Stroth and Unger, 1999; Oudit et al., 2003). ACE is the key enzyme in the generation of Ang II from Ang I, and AngII is a well-known vasoconstrictor that contributes to increased vascular tone and blood pressure (Lonn et al., 1994). Hyperactivity of ACE increases the level of Ang II, which leads to arterial vasoconstriction and glomerular effects, such as promotion of inflammation, hypertrophy, and fibrosis in arteries (Griendling et al., 1993). These effects also contribute to elevating blood pressure and heart rate in the process of hypertension (Lavoie and Sigmund, 2003; Crowley et al., 2006). ACE2 has contradictory actions to ACE. ACE2 metabolizes Ang II into smaller non-hypertensive metabolites, such as Ang (1–7), which is an active substance for lowering blood pressure and reducing heart rate (Ferrario et al., 1997). Multiple studies have indicated that ACE2 is involved in counterbalancing the detrimental effects of Ang II and exerting protective effects, such as anti-fibrosis, antioxidant, and anti-inflammatory effects, within and beyond the cardiovascular system (Donoghue et al., 2000; Crackower et al., 2002; Oudit et al., 2003; Burrell et al., 2004; Ocaranza et al., 2006; Reudelhuber, 2006). Taken together, BAC could directly inhibit the activity and protein expression of ACE and increased the activity of ACE2 to attenuate hypertension.

To clarify the underlying pharmacological mechanism of BAC on anti-hypertension by targeting ACE/ACE2, we further studied the endothelium-dependent vasorelaxation and vascular inflammation in BAC-treated SHRs. Endothelium-dependent vasorelaxation (Knox et al., 2019) and vascular inflammation are the most important events in hypertension. The Akt/eNOS signaling pathway is closely related to endothelium-dependent vasorelaxation, and the inflammatory factors are directly related to vascular inflammation. The eNOS phosphorylation induced by Akt is a key event in the production of NO, which is a direct mediator for endothelium-dependent vasorelaxation (Palmer et al., 1988; Bhandari et al., 2006), while the release of TNF-α and IL-6, the activation of COX-2 expression, and IKB-α phosphorylation are a series of marker events of vascular inflammation. As previously reported, inhibition of ACE and activation of ACE2 can promote vasodilation associated with Akt/eNOS signaling pathway and reduce vascular inflammation (Bader et al., 2018; Shi et al., 2019). In our study, we found that BAC increased total serum NO, and activated Akt/eNOS pathway in BAC-treated SHRs, while the level of TNF-α and IL-6, expression of COX-2, and phosphorylation of IKB-α decreased. These findings suggested that BAC could promote endothelium-dependent vasorelaxation and ameliorate vascular inflammation to attenuate hypertension by inhibiting ACE and activating ACE2.

## Conclusion

BAC targets ACE/ACE2 to enhance endothelium-dependent vasorelaxation and reduce vascular inflammation to attenuate hypertension as a potential modulator of the renin–angiotensin system.

## Data Availability

The original contributions presented in the study are included in the article/Supplementary Materials, further inquiries can be directed to the corresponding author.

## Abbreviations

ACEIs, angiotensin-converting enzyme inhibitors; Ang, angiotensin; ARBs, Ang II receptor blockers; BAC, benzoylaconitine; BMC, benzoylmesaconine; BHC, benzoylhypaconitine; CCK-8, Cell Counting Kit-8; DAPI, 4′,6-diamidino-2-phenylindole dihydrochloride; DBP, diastolic blood pressure; EC50, 50% effective concentration; ECM, endothelial cell medium; HUVECs, human umbilical vein endothelial cells; IC50, 50% inhibiting concentration; LD50, median lethal dose; MAP, mean arterial pressure; MDAs, monoester-diterpenoid alkaloids; RAS, renin–angiotensin system; SHRs, spontaneously hypertension rats; SD rats, Sprague-Dawley rats; DMSO, dimethyl sulfoxide; SPR, surface plasmon resonance; rhACE, recombinant human ACE; PBS, phosphate buffered saline; SBP, systolic blood pressure; UHPLC-MS/MS, ultra-high-performance liquid chromatography-tandem mass spectrometry.
