# Supplementary figures and images for: A Novel Modulator of the Renin–Angiotensin System, Benzoylaconitine, Attenuates Hypertension by Targeting ACE/ACE2 in Enhancing Vasodilation and Alleviating Vascular Inflammation
Source: Front Pharmacol. 2022 Mar 11;13:841435. doi: 10.3389/fphar.2022.841435 (PMC8963105; doi:10.3389/fphar.2022.841435)

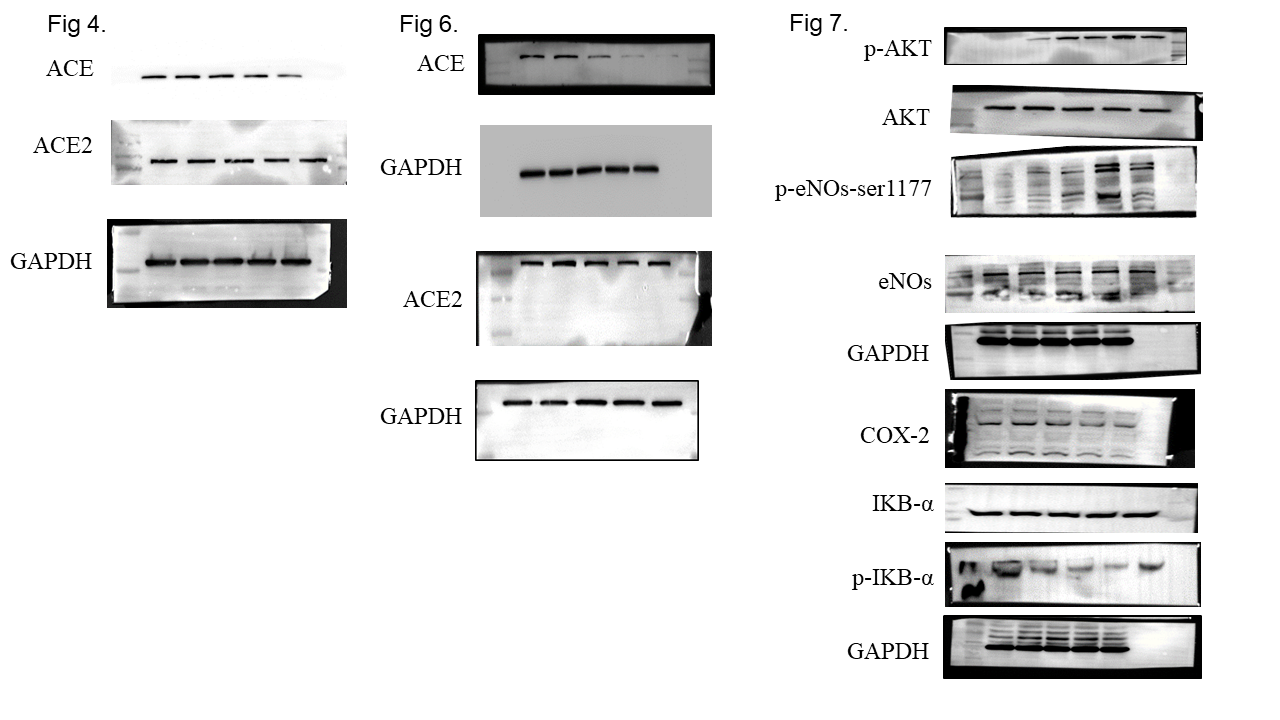

Supplement: Supplementary file 1 [file Image2.TIF]

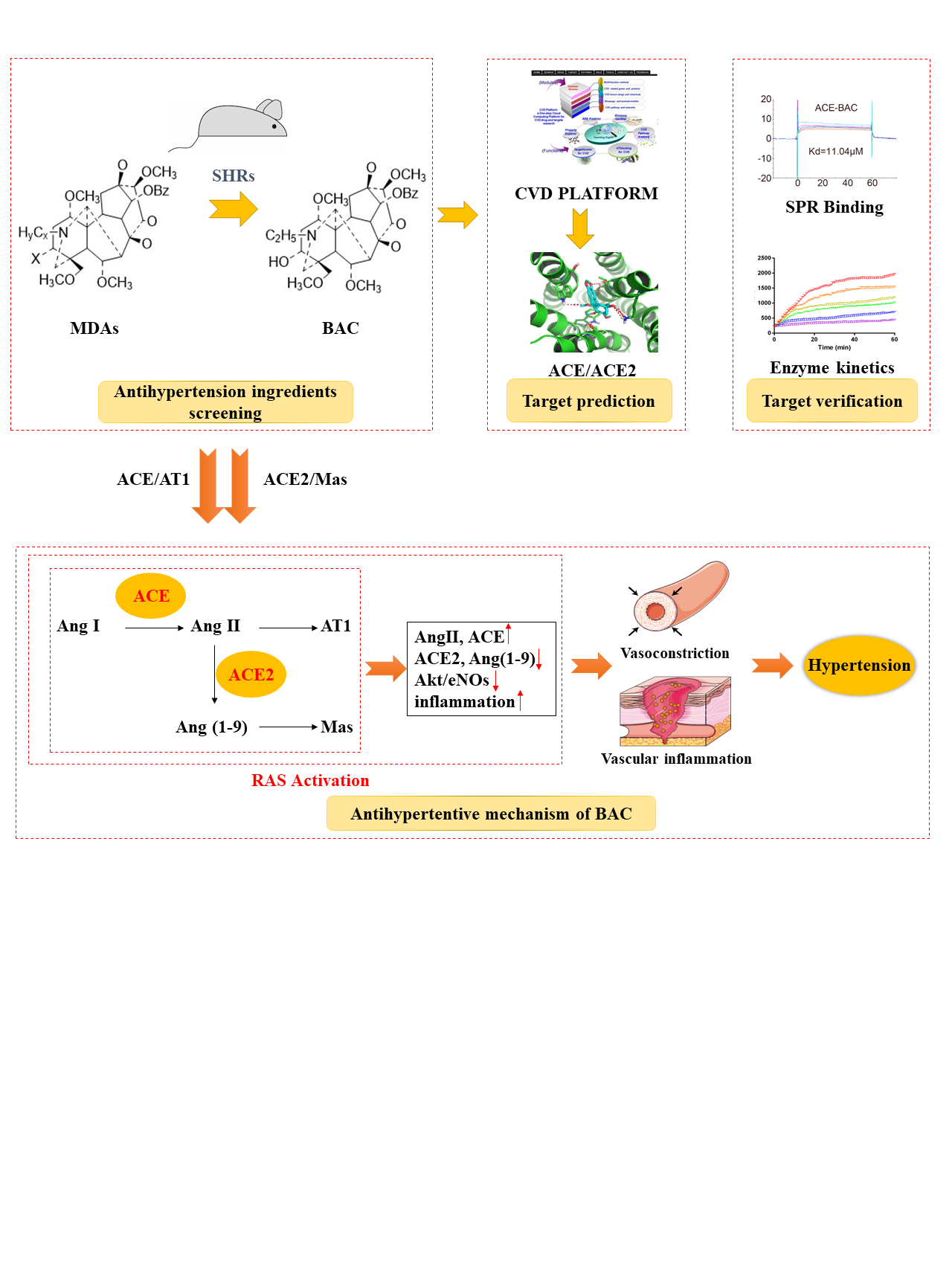

Supplement: Supplementary file 2 [file Image1.TIF]
